# Clinical Performance of the Bioperio^®^ Protocol to Manage Periodontitis

**DOI:** 10.3390/jcm14051738

**Published:** 2025-03-04

**Authors:** Filippo Graziani, Enrica Conticini, Laura Bettini, Greta Ciardelli, Serena Leuci, Crystal Marruganti, Rossana Izzetti

**Affiliations:** 1 Department of Surgical, Medical and Molecular Pathology and Critical Care Medicine, University of Pisa, 56126 Pisa, Italy; 2 Private Practice, 56126 Pisa, Italy; 3 Department of Medical Biotechnologies, University of Siena, 53100 Siena, Italy

**Keywords:** dental enamel proteins, dental scaling, oral hygiene, periodontal treatment, periodontitis, subgingival curettage

## Abstract

**Objectives**: The aim of this study was to describe the structure and assess the efficacy of a patient-centered framework for managing periodontitis, utilizing the Bioperio^®^ protocol, a standardized treatment approach incorporating both clinical and extra-clinical phases. **Methods**: Patients diagnosed with periodontitis were included in this multicenter, single-arm, clinical observational study with a 3-month follow-up. All patients were treated following the Bioperio^®^ protocol, involving professional supra-gingival scaling, oral hygiene instructions, and scaling and root planing following a full-mouth approach. In Stage III/IV periodontitis cases, enamel matrix derivatives (EMD) were applied in periodontal pockets > 5 mm. Monthly recalls were performed until the 3-month follow-up. **Results**: In total, 663 patients were enrolled, with 76.4% being diagnosed with Stage II/III periodontitis. At 3 months, all clinical periodontal parameters improved regardless of the initial stage of periodontitis, achieving pocket closure in 75.4% of cases and patient resolution in 91.3% of the sample. Stages I/II showed significantly improved outcomes compared to Stage IV. The adjunct of EMDproved beneficial, especially in stage III patients, increasing pocket closure by 15% and doubling the odds of patient resolution. No adverse effects of the treatment protocol were observed throughout the study. **Conclusions**: The Bioperio^®^ protocol appears to be a safe and effective therapeutic approach for the management of patients affected by periodontitis. Combining a stepwise approach for clinical phases with tailored oral hygiene instructions and motivational sessions offers a comprehensive strategy that may enhance outcomes for patients with periodontitis.

## 1. Introduction

Periodontitis is a chronic inflammatory disease affecting the supporting structures of the teeth, including the gingiva, periodontal ligament, and alveolar bone [1]. It is primarily caused by the accumulation of pathogenic biofilm (plaque) that triggers an immune response, leading to tissue destruction [2]. Key mechanisms include excessive production of pro-inflammatory cytokines (e.g., IL-1β and TNF-α) and matrix metalloproteinases (MMPs), which degrade the extracellular matrix [3]. The disease progresses through the release of inflammatory mediators, such as cytokines and enzymes, which result in the breakdown of connective tissue and alveolar bone resorption [3]. Local release of cytokines is also triggering a systemic inflammatory response that is shown to be chronically elevated in periodontitis patients [4]. This, together with frequent bacteriaemia, is the basis of the systemic impact of periodontitis [5]. Periodontitis has been associated with more than 50 systemic diseases [6]. Indeed, subjects with periodontitis show inferior glycemic control and are more prone to developing incident diabetes [7]. Additionally, cardiovascular disease and osteoporosis have been linked to periodontal inflammation, highlighting the systemic impact of periodontitis [8].

Clinically, periodontitis is characterized by several distinct features, including gingival inflammation, periodontal pocket formation, and attachment loss [9]. Common signs include bleeding on probing, increased probing depth, gingival recession, and the presence of purulent exudate [10]. In more advanced stages, patients may experience tooth mobility, drifting, and eventual tooth loss if the condition is left untreated [11]. Additionally, radiographic examinations, particularly periapical and panoramic X-rays, often reveal alveolar bone loss, a hallmark of periodontitis lesions [12]. These combined approaches improve diagnostic accuracy and aid in tailoring personalized treatment strategies.

The 2017 classification system for periodontitis introduced a staging and grading framework to improve diagnosis and treatment planning [13,14,15]. Staging defines the severity and extent of periodontitis based on clinical attachment loss (CAL), probing depth (PD), radiographic bone loss, tooth loss due to periodontitis, and complexity factors such as furcation involvement or ridge defects. It is divided into four stages: Stage I (initial), Stage II (moderate), Stage III (severe with potential for additional tooth loss), and Stage IV (advanced with extensive tooth loss and functional impairment) [3]. Severity is determined primarily by CAL and radiographic bone loss, while complexity considers factors that may affect management [3]. Grading assesses the rate of disease progression, the risk of future progression, and the influence of systemic or environmental factors. It is categorized into Grade A (slow progression), Grade B (moderate progression), and Grade C (rapid progression) [3]. Grading is determined by factors such as radiographic bone loss over five years, smoking status, and the presence of diabetes [3]. This system allows for a more personalized approach to periodontal diagnosis and treatment, considering both current disease status and potential future risk [15].

Current therapeutic protocols for periodontitis follow a stepwise approach aimed at controlling infection, reducing inflammation, and preventing disease progression [15,16]. The stepwise approach in periodontal treatment is a structured, evidence-based methodology developed according to the European Federation of Periodontology (EFP) guidelines, that guides the sequence of interventions for managing periodontal disease in stages, focusing on disease control and patient-centered care [16]. This protocol prioritizes initial, conservative methods to reduce biofilm and calculus accumulation, followed by an evaluation phase to determine the necessity of more invasive treatments, such as surgical interventions [17]. The sequential step by step approach provides a thorough assessment of patient response, allowing clinicians to adjust treatment based on individual needs and outcomes [18].

Initial therapy (Steps 1–2, behavioral and mechanical therapy) focuses on patient education, risk factor modification (e.g., smoking cessation, diabetes control), and non-surgical periodontal therapy, including scaling and root planing (SRP) to remove bacterial biofilm and calculus. These methods aim to reduce bacterial load and decrease inflammation, addressing the primary etiological factors of periodontal disease [19]. Studies have shown that this initial therapy can significantly improve periodontal parameters [20,21]. SRP alone is considered the gold standard treatment and can achieve substantial clinical improvements by reducing probing depth and bleeding on probing, essential indicators of periodontal health [22,23,24]. Step 3 (surgical therapy) is indicated for patients with residual deep pockets after Steps 1 and 2 and it involves flap surgery, regenerative procedures (e.g., guided tissue regeneration, bone grafting), or resective surgery to reshape or remove diseased tissues [16]. The final phase of the treatment (Step 4, supportive periodontal therapy) involves a periodontal maintenance program with frequent recalls to prevent recurrence.

One of the significant advantages of the stepwise approach is its emphasis on re-evaluation following non-surgical therapy [25]. Reassessing the patient’s periodontal status after initial interventions allows clinicians to identify residual inflammation or pockets that may require further treatment [26,27]. This intermediate assessment ensures that surgical interventions, such as open-flap debridement or regenerative procedures, are only employed when strictly necessary, thus reducing patient exposure to unnecessary surgical risks and complications [28]. By controlling inflammation beforehand, the surgical phase, if needed, is performed on a healthier foundation, which enhances wound healing and reduces postoperative complications [29]. Another benefit of the stepwise approach is its flexibility and adaptability to different stages of periodontal disease progression [30]. This patient-centered approach considers individual variability, thereby promoting long-term stabilization of periodontal health and reducing the likelihood of disease recurrence [31]. Through regular reassessment and personalized care, clinicians can better monitor and manage disease activity, adjusting the treatment plan as needed to prevent future progression [32].

Furthermore, the stepwise approach has been associated with improved patient compliance and satisfaction [33]. The gradual progression from less invasive to more intensive therapies allows patients to experience improvements early on, which can boost their motivation to adhere to recommended oral hygiene practices [34]. This structured strategy also aligns with the principles of minimally invasive dentistry by prioritizing non-surgical over surgical solutions, whenever possible, leading to better cost-effectiveness and long-term management of periodontal diseases [35].

In the light of the advantages offered by the stepwise approach, a novel therapeutic protocol (Bioperio^®^ protocol) was devised to create an organized, patient-centered framework for the treatment of patients affected by periodontitis using a standardized treatment protocol based on both clinical and extra-clinical phases. The Bioperio^®^ protocol is characterized as a minimally invasive and personalized approach to treating periodontitis, aiming at reducing the need for surgical interventions. Through an initial phase of clinical evaluation and detailed diagnosis, the patient is educated on proper oral hygiene, a key element for therapeutic success (extra-clinical phase). The clinical treatment allows for the effective removal of plaque and calculus both supra- and sub-gingivally, promoting tissue healing in about three months. The protocol includes constant monitoring and a gingival refinement process to optimize results, avoiding surgery in moderate and severe cases. Once periodontal health is stabilized, the supportive therapy program ensures long-term maintenance with regular check-ups and professional cleaning treatments. These strategies together ensure an effective and lasting treatment, promoting tissue regeneration and prolonging the life of the teeth.

The aim of the present study was therefore to assess the 3-month performance of the Bioperio^®^ protocol.

## 2. Materials and Methods

### 2.1. Study Design

This is a multicenter, single-arm, prospective clinical observational study with a 3-month follow-up, conducted on subjects with periodontitis. The study protocol was approved by the Institutional Review Board of the University of Pisa (Pisa, Italy) (protocol number 30/2023). The study was conducted in seven private practice centers located in Northern and Central Italy. All investigations were carried out according to the principles outlined in the Declaration of Helsinki on experimentation involving human subjects. All patients signed an informed consent form to be included in the study. Eligibility criteria are reported in Table 1. The diagnosis of periodontitis was defined as proximal attachment loss in ≥2 non-adjacent teeth [3].

### 2.2. Examiners and Operators’ Calibration

The baseline clinical evaluation and the treatment procedures were conducted by calibrated examiners, trained at the coordination center. The calibration process involved three evaluation rounds per participant, conducted by the examiner in comparison with an expert reference examiner (FG). During each round of calibration, periodontal measurements were assessed independently for consistency and accuracy on patients who were not part of the study. Practitioners were trained during the calibration process to apply consistent pressure when performing periodontal probing (0.25 N) by practicing on a scale measuring force [36]. Discrepancies between the examiner and the expert were reviewed and addressed through feedback and additional training. Five examiners were trained for this cohort.

### 2.3. Baseline Examination

Patients underwent an initial clinical evaluation aimed at assessing the presence of periodontitis. Along with periodontal parameters, complete medical and dental histories were recorded. Information on smoking status (non-smoker, current smoker, or ex-smoker) was registered.

All participants underwent a comprehensive periodontal examination at baseline. The examination protocol involved the registration of periodontal parameters, including probing pocket depth (PPD) and gingival recession (REC), measured across the full mouth at six sites per tooth using a periodontal probe (UNC 15, Hu-Friedy, Chicago, IL, USA). Clinical attachment level (CAL) was determined by adding PPD and REC values. Measurements were rounded to the nearest millimeter. Bleeding on probing was recorded dichotomously at six sites per tooth, allowing for the calculation of full-mouth bleeding score (FMBS) following the methodology described by Lang et al. (1975) [37]. All patients underwent radiographic examination at baseline to evaluate staging and grading [15].

### 2.4. The Bioperio^®^ Protocol

The Bioperio^®^ protocol was structured in accordance with the stepwise approach described by the EFP guidelines [8,16], thus involving an initial phase of patient instruction and motivation followed by supra and sub-gingival instrumentation. In all patients, professional supra-gingival scaling with ultrasonic instruments and air-flow polishing were performed (Step 1) [38]. No intentional instrumentation of the gingival crevice/sulcus was carried out at this phase. Patients received oral hygiene instructions (OHIs), and motivation was provided to improve plaque control. Patients were also instructed to use a rotating oscillating toothbrush (Oral-B^®^, Procter & Gamble, Cincinnati, OH, USA) and inter-proximal brushes (TePe^®^, Malmö, Sweden); OHIs were reinforced during each treatment session and study follow-up.

**Figure 1:**
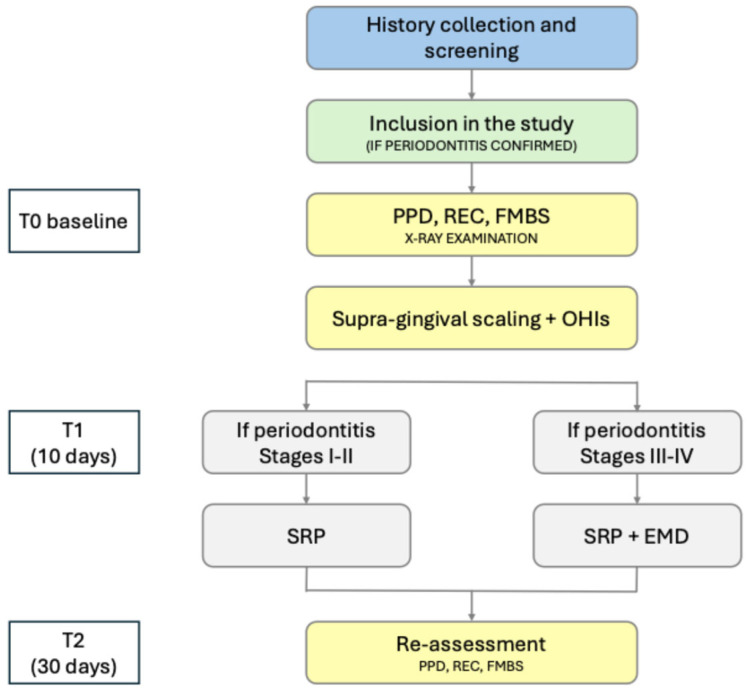
The Bioperio^®^ protocol flowchart indicating the steps of treatment.

One week after Step 1, subgingival mechanical instrumentation was performed (Step 2). Root surfaces were instrumented with mini-micro curettes (Hu-Friedy, Chicago, IL, USA) and ultrasonic instrumentation with periodontal fine tips (EMS, Nyon, Switzerland) under magnification by the examiner. Local anesthesia was administered when needed. Treatment sessions followed a full-mouth approach (FM-SRP) [25]. In cases of Stage III and IV periodontitis, sites with deep PPD (>5 mm) were treated with subgingival mechanical instrumentation with the application of enamel matrix derivatives (EMD) (Emdogain^®^ FL, Institut Straumann AG, Basel, Switzerland) as already been described [22]. Patients were then reassessed three months (90 days) after subgingival instrumentation. During this period, patients attended monthly recalls for plaque control and polishing. The study protocol is depicted in Figure 1.

### 2.5. Outcome Assessment

At baseline, a calibrated investigator performed the initial assessment with the history registration and clinical evaluation. History registration involved recording smoking habits (current, former, or non-smoker). Patients then underwent periodontal charting and x-ray examinations. Periodontal charting was then repeated 90 days after the session of subgingival instrumentation by the same examiner. Parameters collected in the charting were PPD, REC, CAL, and FMBS, as explained in Section 2.3.

### 2.6. Statistical Analyses

All analyses were performed through ad hoc statistical software (STATA BE, version 17.1, StataCorp LP, College Station, TX, USA). Continuous variables were expressed as mean ± standard deviation (SD), while categorical variables were expressed as the number of observations (percentage, %). The normality of data distribution was checked through visual inspection and the Shapiro–Wilk test. After verification of data distribution, simple regression models were used to calculate pre-post differences were expressed using the mean differences (MD) with 95% Confidence Interval (95% CI). Simple and multiple linear/logistic regression models were used to assess the association between periodontitis Stage and pocket closure and pocket resolution, respectively. Multiple (adjusted) models were built using sex, body mass index, smoking, type of diet, and physical activity as confounders. Finally, the same models were employed to compare the effect of SRP vs. SRP + EMD on the pocket closure and patient resolution after periodontal therapy in participants affected by Stage III and Stage IV periodontitis.

## 3. Results

### 3.1. Study Population and Power Calculation

The final sample comprised 663 patients (55.1% females), with a mean age of 46.9 years. Most of the patients were non-smokers, and the mean BMI was 24.3. In terms of periodontal health, Stages II and III were the most frequently diagnosed, accounting for 76.4% of the sample. Grades B and C were the most common among the study samples (Table 2). A post hoc power calculation was performed; the mean proportions of pockets closed in the reference [39] (62.4%) and in the current study (75.4%) were considered. With an alpha error of 5%, the power was over 90%.

### 3.2. Side Effects

No side effects were reported after treatment. Only a few patients reported a minor increase in dental hypersensitivity, which lasted only a few weeks as reported by the clinicians involved. No pain, abscesses or swollen sites were noted after treatment.

### 3.3. Treatment Outcomes

At the 3-month follow-up, the Bioperio^®^ protocol improved all clinical periodontal parameters regardless of the initial stage of periodontitis (Table 3).

Pocket closure was observed in 75.4% of cases, while patient resolution was achieved in 91.3% of patients. When analyzing pocket closure depending on disease staging, Stages I/II achieved significantly higher pocket closure compared to Stage IV (*p* < 0.001). A significant difference (*p* < 0.05) was also observed in terms of patient resolution between Stages I/II and Stage IV (Table 4). A possible inverse dose–response relationship was found between the severity of periodontitis and the reduced proportion of pocket closure and odds of reaching patient resolution (*p* < 0.05).

A subgroup analysis was carried out for Stages III and IV and compared the type of periodontal therapy (SRP vs. SRP + EMD). For both stages, SRP + EMD was associated with significantly higher pocket closure and patient resolution. In Stage III, SRP + EMD was associated with 15.1% higher pockets closure and with 2 times higher odds of achieving patient resolution, even after confounders adjustments (*p* < 0.05). The same trend was noted in cases of Stage IV, although it did not reach statistical significance (Table 5).

## 4. Discussion

The present results suggest that the Bioperio^®^ protocol may effectively treat most of the active sites of periodontitis. The treatment provided a significant improvement in all the periodontal parameters analyzed, which reflects the efficacy of the protocol in treating periodontitis in all its stages. Indeed, although Stages III and IV presented lower rates of resolution and pocket closure at 3 months compared to Stages I–II, it appears worth mentioning that overall results highlight a 75% of pocket closure and 91% of patient resolution. The high rate of pocket closure and patient resolution supports the application of such an approach in treating periodontitis regardless of the disease stage. These data are of importance as the historical comparison with the actual reference of pocket closure—i.e., the meta-analysis that is the basis for the actual EFP guidelines on performance of non-surgical treatment—indicated an expected rate of pocket closure of 58% 3–4 months after non-surgical treatment [20].

The stepwise approach to treating periodontitis has demonstrated effectiveness in addressing the multifactorial nature of this disease [16]. By combining mechanical debridement with patient-centered strategies and adjunctive therapies, such a framework has optimized clinical outcomes while fostering long-term periodontal health and stability [40]. Among the key points, the incremental approach depending on disease stage involves not only the chairside approach to treatment but also the guidance towards a behavioral change, which is fundamental for obtaining long-term patient compliance. The Bioperio^®^ protocol presents some novelties in comparison to traditional protocols. Firstly, there is an extensive attention to Step 1 with, at least, two sessions dedicated to the patient to adapt behaviors to correct plaque control. Subsequently, subgingival instrumentation is performed with minimally invasive instruments and the adjunctive usage of EMD in advanced cases. Further detailed and consecutive care is taken in the post-operative period before examination.

Subgingival instrumentation is widely regarded as effective in reducing PPD and improving CAL, especially when performed with precision and care [41]. The reduction in subgingival bacterial load achieved through SRP plays a critical role in controlling inflammation and halting disease progression [36]. Consistently with these findings, our study observed significant improvements in all clinical periodontal parameters following SRP, reinforcing its role as an essential component of initial therapy. These results highlight the need for thorough execution of mechanical debridement to establish a foundation for subsequent interventions. Our results appeared to be higher than the recommended thresholds achieved after a second session of non-surgical instrumentation [27], as 74% of pocket closure is normally foreseen 6–8 months after 2 sessions of treatment. Another field research study with similar dimensions of the sample showed as well 75% of all pockets resolved in patients with stage II periodontitis and between 66% and 50% in patients with stage III–IV periodontitis, respectively, at 6 months [42]. Our data indicated that these findings can be achieved even 3 months after one sole course of subgingival instrumentation.

However, achieving sustainable improvements requires more than mechanical therapy alone. Motivation and education are critical components in ensuring long-term success [43]. Effective patient education focuses on promoting proper oral hygiene practices, including toothbrushing techniques, interdental cleaning, and plaque control strategies [44]. Studies have consistently shown that patient compliance is a key determinant of treatment outcomes, with regular reinforcement of oral hygiene practices leading to improved clinical parameters over time [45]. Repeated motivational sessions during monthly follow-ups are instrumental in maintaining low full-mouth plaque scores and full-mouth bleeding scores, as continuous education and monitoring significantly improve both patient adherence and clinical results [46]. Furthermore, the psychological and behavioral aspects of motivation cannot be understated, as fostering patient engagement ensures they remain active participants in their periodontal care journey [47].

The Bioperio^®^ protocol comprises the adjunct application of EMD in stage III and IV when PPD of at least 6 mm is present. The enhancement of the outcomes of non-surgical periodontal therapy with EMD is well established [25]. EMD is known for its regenerative properties, promoting the formation of new cementum, periodontal ligament, and alveolar bone, particularly in sites with intrabony defects [48,49,50]. Numerous studies have shown that combining EMD with SRP provides superior CAL gains and greater PPD reductions compared to SRP alone, especially in cases involving advanced defects [51,52,53]. Our findings align with previous observations, with sites treated using EMD showing improved healing and better periodontal outcomes compared to those treated solely with SRP, possibly through mechanisms of wound healing enhancement via blood clot stabilization as our group already suggested [25]. Clearly, the odds of achieving patient resolution and pocket closure were increased with the adjunctive usage of EMD.

The authors are aware of the intrinsic limitations of the present study. These data are the result of data collection of a clinical protocol shared among different clinical practices. Therefore, the actual study presents the typical limitations of a field research study [54,55]. Firstly, no comparison with an overall control group was provided as the study. However, comparison on sub-groups could be made (i.e., the adjunctive use of EMD in Stages III–IV). The calibration of the examiners focused on pockets and attachment loss. Therefore, the initial patient sample undergoing treatment did not have a plaque score registered, and patient motivation was not evaluated throughout the study. Moreover, no calibration on bleeding or probing was provided, thus limiting the evaluation of the protocol in terms of efficacy in inflammation control. The population was representative of incoming patients of a dental practice and smokers were included in the study sample, potentially impacting the final results. Yet, smoking was one of the factors included in the statistical adjustment. The short-term follow-up may also represent a limitation to the present study, however, in line with the vast majority of current literature. The discrepant number in the periodontitis cases depending on the stage might have skewed the results, as only a minority of patients were eventually affected by Stage IV disease. Nevertheless, the vast extent of the sample represents a unicum in periodontal literature contributing to the clinical significance of the data.

This study offers valuable insights into the potential role of the Bioperio^®^ protocol in the management of patients with periodontal disease. The findings highlight the protocol’s efficacy in achieving significant clinical improvements, including a high rate of disease resolution and pocket closure within a three-month follow-up period. These results underscore its effectiveness in reducing probing pocket depths and controlling inflammation. Furthermore, the Bioperio^®^ protocol’s structured, stepwise approach may contribute to its success by addressing both the microbial and host-related factors involved in periodontitis. By incorporating tailored mechanical debridement and potentially adjunctive therapies, the protocol not only facilitates pocket closure, but also creates a stable periodontal environment conducive to long-term maintenance. These outcomes align with prior research supporting the importance of targeted interventions and patient adherence in achieving sustained clinical benefits [56].

Future studies with longer follow-up periods could further elucidate the long-term oral and systemic benefits and broader applicability of the Bioperio^®^ protocol. Additionally, comparative analyses with other treatment modalities could provide deeper insights into its unique advantages, helping to optimize periodontal care strategies.

## 5. Conclusions

The Bioperio^®^ protocol provides a comprehensive and adaptable framework for managing periodontitis and appears effective as observed in our results on a large cohort of periodontitis affected patients. Despite the limitations of the present study, it appears that this strategy might address all the various stages of disease with relevance. Further studies to validate and refine this protocol are advised, potentially exploring the optimal timing, sequencing, and motivation of the patients through plaque control and combinations of treatments to enhance efficacy and predictability in diverse patient populations.

## Figures and Tables

**Table 1:** Eligibility criteria for participation in the study.

Inclusion Criteria	Exclusion Criteria
Patients > 18 years of age	Age < 18 years
Confirmed diagnosis of periodontitis	Pregnancy or breastfeeding
No previous treatment of periodontitis	Patients previously treated for periodontitis
Systemic health or well-controlled chronic medical conditions	Poorly controlled systemic conditions
Willingness to participate in the study	Refusal to participate in the study
Compliance with study follow-up	Patients who did not complete the proposed treatment plan

**Table 2:** Baseline characteristics of study population. Data are expressed as mean (SD) or N (%).

Variable	Overall
Age	46.9 years (±33.1)
Gender	298 males (44.9%)
	365 females (55.1%)
BMI	24.3 (±6.3)
Smoking habit	167 Smokers (25.2%)
	302 Non-smokers (45.6%)
	194 Former smokers (29.2%)
Type of diet	626 Omnivore (94.4%)
	37 others (5.6%)
Physical activity	411 Active (62.0%)
	252 Sedentary (38.0%)
Periodontitis extent (EFP/AAP)	240 Localized (36.2%)
	423 Generalized (64.8%)
Periodontitis stage (EFP/AAP)	Stage I 105 (15.8%)
	Stage II 298 (45.0%)
	Stage III 209 (31.5%)
	Stage IV 51 (7.7%)
Periodontitis grade (EFP/AAP)	Grade A 66 (10.0%)
	Grade B 361 (54.4%)
	Grade C 236 (35.6%)

Abbreviations: BMI, body mass index; EFP/AAP: European Federation of Periodontology/American Academy of Periodontology.

**Table 3:** Periodontal parameters before and after treatment.

Variable	Timepoint	Mean (SD)			
		Overall	Stages I/II	Stage III	Stage IV
N. pockets ≥ 4 mm	Baseline	39.3 (23.5)	28.4 (12.3)	50.6 (25.8)	65.9 (31.5)
	Day90	1.5 (4.4)	1.0 (3.1)	2.2 (5.0)	3.6 (8.5)
	*p*-value intragroup	<0.001	<0.001	<0.001	<0.001
MD (95% CI) pre-post		37.8 (35.1, 40.51) ***	27.4 (23.8, 30.9) **	48.4 (45.4, 51.4) ***	62.3 (58.6, 65.9) ***
N. pockets ≥ 5 mm	Baseline	25.1 (20.7)	15.6 (7.4)	35.1 (21.9)	51.2 (30.9)
	Day90	0.6 (2.8)	0.6 (2.2)	1.0 (3.6)	0.5 (1.5)
	*p*-value intragroup	<0.001	<0.001	<0.001	<0.001
MD (95% CI) pre-post		24.5 (22.1, 26.9) ***	15.0 (12.8, 17.2) ***	34.1 (31.6, 36.6) **	50.7 (47.2, 54.2) ***
N. pockets ≥ 6 mm	Baseline	7.1 (11.8)	1.8 (2.9)	13.0 (12.4)	23.8 (20.3)
	Day90	0.3 (1.3)	0.08 (0.6)	0.5 (1.7)	0.7 (2.0)
	*p*-value intragroup	<0.001	<0.001	<0.001	<0.001
MD (95% CI) pre-post		6.8 (5.5, 8.1) ***	1.7 (0.9, 2.6) ***	12.5 (11.1, 13.9) **	23.1 (20.8, 25.4) ***
N. sites CAL ≥ 4 mm	Baseline	30.9 (24.2)	18.9 (13.2)	41.2 (24.2)	64.0 (35.2)
	Day90	6.4 (13.2)	4.5 (5.3)	6.6 (11.1)	18.1 (27.3)
	*p*-value intragroup	<0.001	<0.001	<0.001	<0.001
MD (95% CI) pre-post		24.5 (21.4, 27.6) ***	14.4 (10.4, 18.4) **	34.6 (31.6, 37.6) ***	45.9 (40.8, 50.9) ***
N. sites CAL ≥ 5 mm	Baseline	17.7 (9.6)	16.9 (8.2)	18.2 (9.9)	15.7 (9.6)
	Day90	6.9 (11.0)	6.3 (10.9)	7.9 (12.2)	8.7 (9.9)
	*p*-value intragroup	<0.001	<0.001	<0.001	<0.001
MD (95% CI) pre-post		10.8 (9.1, 12.5) ***	10.6 (6.8, 14.4) ***	10.3 (8.5, 12.1) ***	7.0 (5.4, 8.6) ***
% PPD ≥ 4 mm	Baseline	25.7 (15.8)	18.1 (8.7)	32.3 (16.0)	49.2 (20.6)
	Day90	1.0 (2.9)	0.6 (1.9)	1.4 (3.1)	2.8 (5.9)
	*p*-value intragroup	<0.001	<0.001	<0.001	<0.001
MD (95% CI) pre-post		24.7 (22.9, 26.5) ***	17.5 (15.0, 19.9) ***	30.9 (29.1, 32.7) ***	46.4 (43.9, 48.8) **
% PPD ≥ 5 mm	Baseline	16.4 (13.8)	9.5 (6.4)	22.2 (13.5)	37.9 (20.9)
	Day90	0.4 (1.7)	0.3 (1.5)	0.7 (2.2)	0.4 (1.3)
	*p*-value intragroup	<0.001	<0.001	<0.001	<0.001
MD (95% CI) pre-post		16.0 (14.4, 17.6) *	9.2 (7.4, 11.0) **	21.5 (19.9, 23.1) **	37.5 (35.1, 39.9) ***
% PPD ≥ 6 mm	Baseline	4.6 (7.8)	0.9 (1.7)	8.2 (7.5)	17.5 (14.1)
	Day90	0.2 (0.9)	0.05 (1.0)	0.3 (1.1)	0.6 (1.7)
	*p*-value intragroup	<0.001	<0.001	<0.001	<0.001
MD (95% CI) pre-post		4.4 (3.5, 5.3) ***	0.9 (0.2, 1.5) **	7.9 (7.0, 8.8) **	16.9 (15.3, 18.5) ***
% CAL ≥ 4 mm	Baseline	11.4 (6.3)	10.7 (5.7)	11.6 (6.3)	11.8 (6.4)
	Day90	4.7 (7.6)	4.2 (7.5)	5.1 (7.8)	7.1 (7.6)
	*p*-value intragroup	<0.001	<0.001	<0.001	<0.001
MD (95% CI) pre-post		6.7 (5.6, 7.8) **	6.5 (3.9, 9.1) *	6.5 (5.4, 7.6) **	4.7 (3.6, 5.8) ***
% CAL ≥ 5 mm	Baseline	20.5 (16.8)	12.3 (8.3)	26.4 (15.2)	47.6 (23.5)
	Day90	4.8 (10.2)	3.2 (7.9)	4.6 (7.9)	15.5 (21.8)
	*p*-value intragroup	<0.001	<0.001	<0.001	<0.001
MD (95% CI) pre-post		15.7(13.5, 17.9) **	9.1 (5.9, 12.3) ***	21.8 (19.9, 23.7) **	32.1 (28.5, 35.7) ***
FMBS (%)	Baseline	44.2 (19.1)	38.9 (15.2)	47.9 (20.5)	58.9 (23.8)
	Day90	3.1 (6.0)	0.02 (0.05)	0.03 (0.06)	0.06 (0.1)
	*p*-value intragroup	<0.001	<0.001	<0.001	<0.001
MD (95% CI) pre-post		41.1 (38.8, 43.4) ***	38.8 (34.6, 43.1) **	47.9 (45.6, 50.2) ***	58.8 (56.1, 61.5) **

Abbreviations: BMI, body mass index; CAL, clinical attachment loss; FMBS, full-mouth bleeding score; mm, millimeter; N, number of observations; PPD, probing pocket depth; SD, standard deviation; %, percentage. * *p* < 0.05; ** *p* < 0.01; *** *p* < 0.001.

**Table 4:** Periodontal outcomes after treatment steps I–II.

Variable	Timepoint	Mean (SD)							
		Overall		Stage I/II	Stage III	Stage IV			*p*-Value
Pocket closure, %	Day 90	75.4 (15.1)		82.5 (8.9)	69.2 (15.7)	53.6 (19.1)			<0.01
Patient resolution, N (%)	Day 90	605 (91.3)		386 (95.8)	178 (85.2)	41 (80.4)			0.03
Exposure variable	Pocket closure—MD (95% CI)				Patient resolution—OR (95% CI)				
	Unadjusted		Adjusted		Unadjusted		Adjusted		
Stage III/IV (vs. Stage I/II)	−15.2 (−17.3, −13.2) ***		−14.6 (−18.3, −32.9) ***		0.2 (0.1, 0.8) **		0.3 (0.1, 0.9) **		

Abbreviations: N, number of observations; SD, standard deviation; %, percentage. Adjusted for sex, body mass index, smoking, type of diet, and physical activity. * *p* < 0.05; ** *p* < 0.01; *** *p* < 0.001.

**Table 5:** Comparison of treatment outcomes of SRP vs. SRP + EMD in periodontitis stage III and IV.

Variable	Stage III				Stage IV			
	SRP		SRP + EMD	*p*-Value	SRP		SRP + EMD	*p*-Value
Pocket closure, %	65.8 (14.2)		82.2 (14.1)	0.01	51.1 (18.0)		66.5 (20.9)	0.03
Patient resolution, N (%)	138 (93.1)		40 (93.0)	0.07	6 (75.0)		35 (81.4)	0.04
	Pocket closure—MD (95% CI)				Patient resolution—OR (95% CI)			
Exposure variable	Stage III		Stage IV		Stage III		Stage IV	
	Unadjusted	Adjusted	Unadjusted	Adjusted	Unadjusted	Adjusted	Unadjusted	Adjusted
SRP + EMD (vs. SRP)	16.4 (11.6, 21.2) ***	15.1 (10.2, 23.1) **	15.4 (10.9, 19.9) **	14.2 (9.9, 20.8) **	2.7 (1.9, 8.9) **	2.2 (1.2, 9.1) *	1.8 (1.1, 6.9) *	1.2 (0.8, 9.7)

Abbreviations: N, number of observations; SD, standard deviation; %, percentage. Adjusted for sex, body mass index, smoking, type of diet, and physical activity. * *p* < 0.05; ** *p* < 0.01; *** *p* < 0.001.

## Data Availability

Dataset available upon request from the authors.
